# Clinical Impact of the Expanded BioFire Blood Culture Identification 2 Panel in a U.S. Children’s Hospital

**DOI:** 10.1128/spectrum.00429-21

**Published:** 2021-08-25

**Authors:** Kelly E. Graff, Claire Palmer, Toraj Anarestani, Darcy Velasquez, Stacey Hamilton, Kristin Pretty, Sarah Parker, Samuel R. Dominguez

**Affiliations:** a University of Colorado School of Medicine, Department of Pediatrics, Aurora, Colorado, USA; b Children’s Hospital Colorado, Department of Laboratory and Pathology Medicine, Aurora, Colorado, USA; Children’s Hospital Los Angeles, University of Southern California

**Keywords:** antibiotic resistance, antimicrobial stewardship, blood culture, diagnostics, multiplex PCR, pediatrics

## Abstract

The BioFire blood culture identification (BCID) panel decreases time to pathogen identification and time to optimal antimicrobial therapy. The BioFire blood culture identification 2 (BCID2) panel is an expanded panel with 17 additional targets and resistance genes; however, there are limited data on its impact in pediatric patients. We compared the BioFire BCID2 panel and the BCID panel by assaying BCID2 simultaneously with the current standard of care on 191 consecutive blood culture specimens at Children’s Hospital Colorado. The primary outcome was equivalence, measured as percent agreement between the two panels and standard culture. The theoretical reduction in time to optimal therapy was calculated overall, with subanalyses performed on *Enterococcus* species and Gram-negative resistance genes. The percent agreement was equivalent between the two panels, with BCID at 98% (95% confidence interval [CI], 95 to 100%) and BCID2 at 97% (95% CI, 93 to 99%); the difference was 1.2% (95% CI, −0.8, 3.1%; *P* < 0.0001). There was not a significant reduction in time to theoretical optimal therapy with BCID2 compared to BCID for all cultures (reduction of 9 h, *P* = 0.3). Notably, 13 Enterococcus faecalis isolates were detected on BCID2, which would have resulted in a theoretical reduction in time to optimal antimicrobial therapy of 34 h (*P* = 0.0046). Five CTX-M genes were detected for enteric bacteria. The BioFire BCID2 panel had equal rates of detection compared to the BioFire BCID panel in pediatric patients. It had the advantage of detecting more organisms at the species level, and significantly reducing time to theoretical optimal antimicrobial therapy for Enterococcus faecalis. With the additional resistance genes, it also has the potential to impact care with earlier identification of resistant enteric pathogens.

**IMPORTANCE** The BioFire BCID2 panel is an accurate panel that is equivalent to the BioFire BCID panel compared to standard culture. The BioFire BCID2 panel offers several advantages over the BioFire BCID panel, including enterococcal species identification, Gram-negative resistance gene detection, Salmonella identification, and the added *mec*A/*mec*C and SCCmec right extremity junction (MREJ) target for better Staphylococcus aureus and coagulase-negative Staphylococcus (CoNS) differentiation. Most importantly, it provides additional clinical impact with the potential to decrease the time to optimal antimicrobial therapy compared to the BioFire BCID panel, with likely further impact at institutions with a higher prevalence of Gram-negative resistance.

## INTRODUCTION

Multiplex PCR assays for bloodstream infections, such as the BioFire blood culture identification (BCID) panel, lead to a significant reduction in time to pathogen identification and optimization of antimicrobial therapy (1–5). A previous study at Children’s Hospital Colorado (CHCO) demonstrated that implementation of the BioFire BCID panel coupled with antimicrobial stewardship decreased the time to optimal therapy by 33.5 h compared to standard culture (2). The BioFire BCID panel has 27 targets, which include Gram-positive, Gram-negative, and yeast pathogens, with 3 antimicrobial resistance genes (6).

The expanded BioFire blood culture identification 2 (BCID2) panel was FDA cleared in 2020. The BioFire BCID2 panel has 43 total targets, with 17 additional targets compared to the BioFire BCID panel. This new assay includes targets for 11 Gram-positive bacteria, 15 Gram-negative bacteria, 7 fungal pathogens, and 10 antimicrobial resistance genes (6, 7). This expanded panel is not yet studied in pediatric patients, and therefore its clinical impact in this population is unknown. This study aimed to evaluate the performance of the BioFire BCID2 panel compared to the BioFire BCID panel with standard culture; we hypothesized that BCID2 was equivalent to BCID. Additional clinical impact of BCID2 over BCID was measured as a secondary outcome.

## RESULTS

### Study population.

Demographic and clinical characteristics are summarized in Table 1. Half of the cohort was male (50%), and the majority identified as white (59%) and not Hispanic (59%), with a median age at blood culture draw of 5 years (interquartile range, 0.6 to 12.9 years; range, 0 to 34 years). Almost all patients were hospitalized (94%), with a median length of stay of 7 days (range, 1 to 161 days). Over half of the patients were admitted to the intensive care unit (ICU) (53%), and 11 patients (6%) died during the admission. A total of 75% of the patients had an underlying medical condition, of which 30% were immunocompromised; 46% of blood draws were from central lines, and 24 cultures (13%) were polymicrobial cultures. A total of 46 cultures (24%) were considered contaminants by the treating team; 31 (67%) were coagulase-negative Staphylococcus.

**TABLE 1:** Demographic and clinical characteristics

Characteristic	No. of patients (*n* = 191)
Gender	
Male	95 (50%)
Female	96 (50%)
Age at blood culture draw (yrs), median (range)	5 (0–34)
Ethnicity	
Hispanic or Latino	58 (30%)
Not Hispanic or Latino	112 (59%)
Unknown or not reported	21 (11%)
Race	
American Indian or Alaska Native	4 (2%)
Asian	4 (2%)
Black or African American	11 (6%)
Native Hawaiian or other Pacific Islander	1 (1%)
White	113 (59%)
More than one race	13 (7%)
Unknown or not reported	21 (11%)
Other	24 (13%)
Admitted	
Yes	180 (94%)
No	11 (5.8%)
Length of stay (days), median (range)	7 (1–161)
Admitted to ICU	
Yes	94 (53%)
No	85 (47%)
Condition at discharge	
Discharged home	160 (84%)
Transfer to another facility	1 (1%)
Deceased	11 (6%)
Not yet discharged at time of data entry	19 (10%)
Underlying medical condition	
Yes	144 (75%)
No	47 (25%)
Type of underlying medical conditions	
Prematurity	32 (22%)
Gastrointestinal	49 (34%)
Pulmonary	38 (26%)
Cardiology	42 (29%)
Malignancy/cancer	26 (18%)
Nephrology	8 (6%)
Genetic/metabolic	23 (16%)
Neurology	27 (19%)
Hematology	15 (10%)
Other	27 (19%)
Immunocompromised	
Yes	43 (30%)
No	101 (70%)
Source of blood culture draw	
Central	87 (46%)
Peripheral	104 (54%)

### BioFire BCID2 panel results.

**FIG 1:**
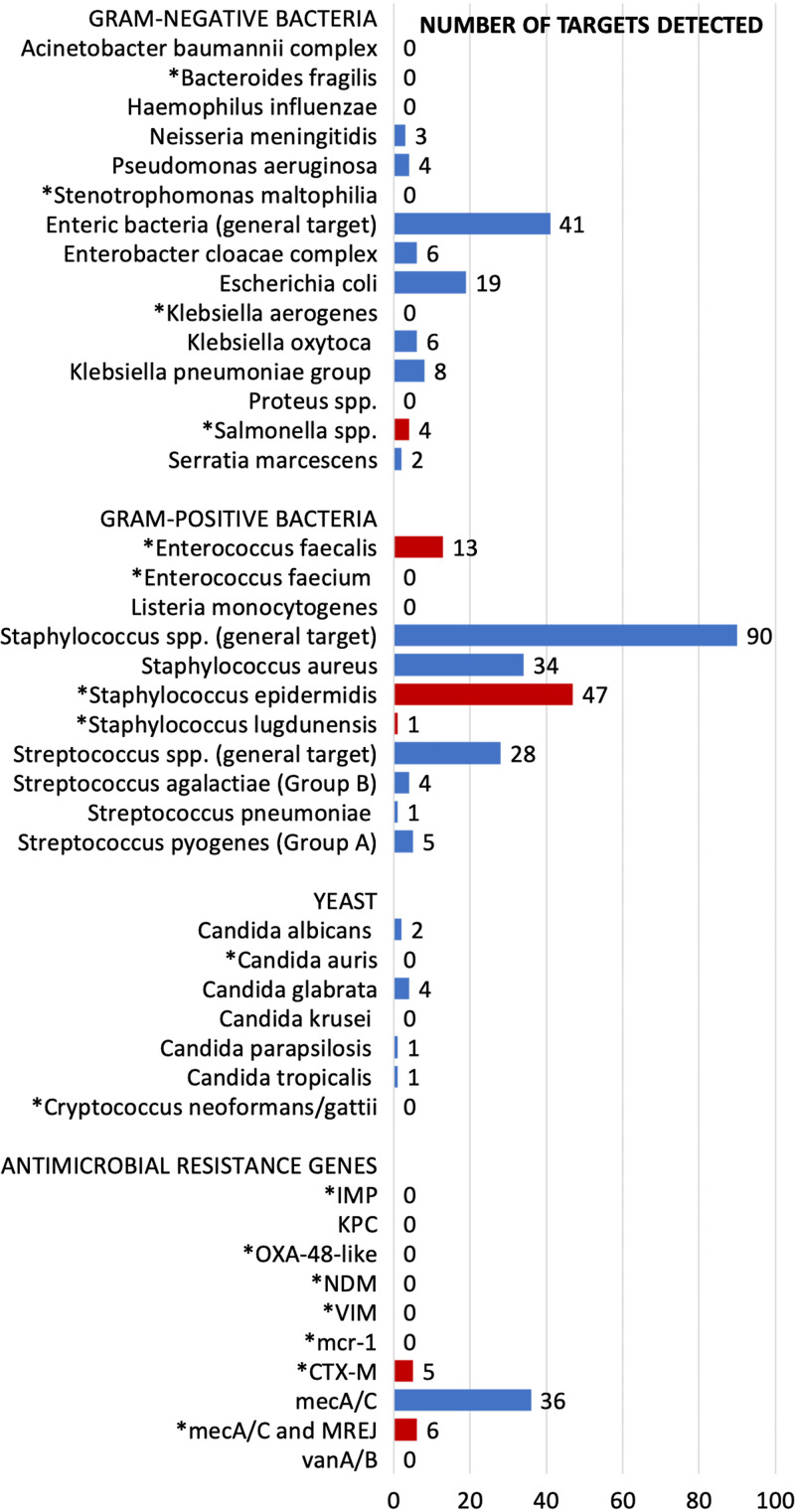
Absolute number of BCID2 targets detected during the study period. Asterisks (*) indicate new targets on the BCID2 panel not previously on the BCID panel. Red bars represent new targets detected on BCID2 during the study. Blue bars represent targets detected on BCID2 and BCID during the study.

Of the 191 samples, 177 (93%) had at least one target detected on both the BCID and BCID2 panels. The most common non-BCID2 targets isolated in culture were *Micrococcus* species, followed by *Rothia* species and *Corynebacteriu*m (see Table S2 in the supplemental material). The most common target detected on the BioFire BCID2 panel was the Staphylococcus species target (*n* = 90, 51%), with 47 (27%) Staphylococcus epidermidis and 34 (19%) Staphylococcus aureus (Fig. 1). A total of 36 (77%) of the Staphylococcus epidermidis targets detected the *mec*A/*mec*C gene; 6 (18%) of Staphylococcus aureus targets detected *mec*A/*mec*C and SCCmec right extremity junction (MREJ), indicating methicillin-resistant Staphylococcus aureus (MRSA). Notably, BCID2 detected 13 (7%) Enterococcus faecalis and 4 (2%) Salmonella species, which were only detected as *Enterococcus* species and *Enterobacterales* targets, respectively, on BCID. No *vanA*/*vanB* targets were detected. Of the Gram-negative resistance genes, 5 (3%) CTX-M genes were detected; no additional Gram-negative resistance genes were detected (Fig. 1). The following new BCID2 targets were never detected: Bacteroides fragilis, Stenotrophomonas maltophilia, Klebsiella aerogenes, Enterococcus faecium, Candida auris, and Cryptococcus neoformans*/*Cryptococcus gattii.

BCID2 correctly identified all target organisms in 97% of culture results (95% confidence interval [CI], 93 to 99%), while BCID correctly identified targets in 98% (95% CI, 95 to 100%) (Table 2). The proportion of BCID2 results that agreed with standard culture was not significantly different from the proportion of BCID results that agreed with standard culture, based on an equivalence margin of 10% and difference of 1.2% (95% CI, −0.8 to 3.1%; *P* < 0.0001). There were 3 BCID results in total that did not match standard culture. All 3 failed to detect a target that grew in standard culture (Enterococcus faecalis [*Enterococcus* target], Staphylococcus hominis [Staphylococcus target], and Citrobacter freundii [*Enterobacterales* target]); all were polymicrobial cultures. There were 5 BCID2 results in total that did not match standard culture. Three failed to detect a target that grew in standard culture (Staphylococcus hominis [Staphylococcus target], Enterococcus faecium, and Citrobacter freundii [Enteric bacteria target]); all were polymicrobial cultures. Two additional cultures had incorrect species identification for coagulase-negative Staphylococcus on the BCID2. (i) BCID2 detected Staphylococcus epidermidis, whereas matrix-assisted laser desorption ionization–time of flight mass spectrometry (MALDI-TOF MS) identified Staphylococcus haemolyticus from standard culture; 16S ribosomal sequencing confirmed Staphylococcus haemolyticus/Staphylococcus devriesei. (ii) BCID2 detected Staphylococcus lugdunensis, whereas MALDI-TOF MS identified Staphylococcus haemolyticus; 16S ribosomal sequencing confirmed Staphylococcus haemolyticus.

**TABLE 2:** Percent agreement and time to event outcomes

Agreement with panel and culture	Proportion with 95% CI	*P* value
BCID	98% (95–100%)	<0.0001^*a*^
BCID2	97% (93–99%)	
**All cultures (*n* = 191)**	**Median hours (IQR)**	
Time to BCID2 result	19 (17–21)	
BCID time to optimal antimicrobial therapy	35 (28–47)	0.3^*c*^
BCID2 time to theoretical optimal therapy^*b*^	26 (22–36)	
***Enterococcus* detected (*n* = 13)**	**Median hours (IQR)**	
BCID time to optimal therapy	51 (35–66)	0.0046
BCID2 time to theoretical optimal therapy	17 (13–21)	
**CTX-M resistance detected (*n* = 5)^*d*^**	**Median hours (IQR)**	
BCID time to optimal therapy^*e*^	20 (11–77)	
BCID time to effective therapy^*f*^	29 (16–40)	
BCID2 time to theoretical optimal therapy	16 (13–18)	

a Primary outcome was equivalence with an *a priori* margin of 10%; difference, 1.2% (95% CI, −0.8–3.1%); a *P* value of <0.05 indicates no significant difference in percentage agreement between the two panels.

b Accounts for the reduction in time for Enterococcus faecalis and CTX-M.

c Comparing the time to optimal therapy and time to theoretical optimal therapy for all cultures.

d Data shown for descriptive purposes; no analysis performed.

e *n* = 3 (two patients never received optimal therapy).

f *n* = 4 (one patient never received effective therapy).

### Time to event outcomes.

Similar to BCID, the median time to BCID2 result was 19 h (95% CI, 17 to 21). The median time to optimal antimicrobial therapy was 35 h (95% CI, 28 to 47) (Table 2). Enterococcus faecalis and the CTX-M gene were the only two BCID2 targets detected that were deemed to result in a quicker time to optimal antimicrobials over BCID (*n* = 18; 9%). After substituting the time to optimal therapy with the time to BCID2 result for these 18 isolates, the overall time to theoretical optimal therapy was 26 h (95% CI, 22 to 36), leading to an overall reduction in time to theoretical optimal therapy of 9 h, which was not statistically significant (*P* = 0.3). Although Salmonella species was a new target detected in this study, all 4 patients were already on optimal therapy at the time of the BCID2 result due to positive multiplex PCR from stool specimens and therefore did not affect the time to theoretical optimal therapy. In a subanalysis with Enterococcus faecalis, assuming that antimicrobial stewardship (ASP) guidelines were acted upon immediately, there was a significant reduction in time to theoretical optimal therapy by 34 h (*P* = 0.0046). Of the 5 patients with the CTX-M gene detected, 2 were from E. coli, 2 were from Klebsiella pneumoniae, and 1 was from Klebsiella oxytoca; all isolates were cephalosporin intermediate or resistant; two patients never received optimal therapy, and one patient died shortly after susceptibilities resulted.

## DISCUSSION

This study demonstrates that the BioFire BCID2 panel is equivalent to the BioFire BCID panel in a U.S. pediatric population. There was no significant difference in percent agreement with standard culture between the two panels, and therefore the primary endpoint was met. The additional two discrepant targets on BCID2 were due to coagulase-negative Staphylococcus misidentification at the species level, which is not clinically meaningful. Overall, the BioFire BCID2 panel is an accurate panel that detects over 90% of all organisms in standard culture.

The BioFire BCID2 panel has several advantages over the BioFire BCID panel. Most notable in our pediatric population was its ability to differentiate *Enterococcus* at the species level. Enterococcus faecalis is 100% susceptible to ampicillin at our institution, and therefore earlier identification would significantly reduce the time to optimal therapy compared to BCID. Importantly, this change would lead to earlier deescalation of vancomycin and potentially less antibiotic adverse effects. It is important to note that BCID2 no longer retains the general *Enterococcus* target. This would result in loss of the ability to identify other *Enterococcus* species, particularly the vancomycin-nonsusceptible species such as E. gallinarum or E. casseliflavus. A previous study conducted at CHCO found that the prevalence of these species among *Enterococcus* bacteremia was 12% (29 isolates) over a 6-year period (8), However, our study only identified one isolate over the 6-month period, which may suggest that the prevalence is declining. Given the much higher prevalence of Enterococcus faecalis and Enterococcus faecium at our institution, the benefit of the BioFire BCID2 panel outweighs the loss of the general *Enterococcus* target from the BioFire BCID panel.

BCID2 has the added benefit of detecting additional Gram-negative resistance genes. The CTX-M gene indicates the presence of extended-spectrum beta-lactamase-producing organisms (9, 10). Identifying this gene earlier would result in a reduction in time to optimal and time to effective therapy. Although we did not detect any carbapenemase-resistant *Enterobacterales* genes (IMP, KPC, OXA-48-like, NDM, VIM, or *mcr-1*), this has a potential added benefit in institutions with higher rates of Gram-negative resistance. In particular, Latin America has carbapenem-resistant *Enterobacteriaceae* (CRE) rates as high as 10%, with many harboring KPC and NDM genes (11–13). Earlier identification of CRE in Latin American countries may lead to a reduction in mortality, but more studies are needed in this population to assess this potential added benefit.

The BioFire BCID2 panel has the ability to differentiate coagulase-negative Staphylococcus (CoNS) species compared to the BioFire BCID panel. In our patient population, the species differentiation does not have a clinical impact and would not affect time to antimicrobials. However, the added *mec*A/*mec*C and MREJ target helps to differentiate mixed cultures containing oxacillin-resistant CoNS and methicillin-susceptible Staphylococcus aureus (MSSA) from those containing methicillin-resistant Staphylococcus aureus (MRSA) (14). This gives more confidence in the reporting of MSSA versus MRSA and the *mec*A/*mec*C target being linked to CoNS. A 5-year analysis of 412 staphylococcal blood culture specimens at CHCO revealed that the absence of *mec*A had 100% concordance with oxacillin susceptibility for both Staphylococcus aureus and CoNS (15). Due to the increased confidence in MRSA and CoNS differentiation, we will now be reporting *mec*A/*mec*C results for Staphylococcus epidermidis and Staphylococcus lugdunensis from the BioFire BCID2 panel and recommending narrowing antimicrobial therapy if *mec*A/*mec*C is negative. This has the potential to provide an additional impact in overall reduction in time to optimal antimicrobial therapy and reduce antibiotic adverse effects.

Lastly, the BioFire BCID2 panel has the added potential benefit of detecting Salmonella species. In our study, all patients already had Salmonella detected by a rapid, multiplex PCR in the stool prior to the BioFire BCID2 panel result and therefore were already on optimal therapy. However, in cases where it is not detected in stool, or at institutions without stool multiplex PCR panels, this could reduce the time to optimal antimicrobials. Although not detected during this study period, the addition of more emerging pathogens, such as Klebsiella aerogenes, *Stenotrophomonas*, and Candida auris, would also be beneficial at institutions where these organisms are increasing in prevalence.

There are limitations with the BioFire BCID2 panel. Both the BioFire BCID and BCID2 panels failed to detect all isolates in polymicrobial cultures; however, they were both able to detect all isolates in 87.5% of polymicrobial cultures. The loss of the general *Enterococcus* target is the main limitation of the BioFire BCID2 panel over the BioFire BCID panel. At our institution, the prevalence of vancomycin-nonsusceptible *Enterococcus* species is low and therefore does not outweigh the benefits of the added identification of *Enterococcus* isolates to the species level. The BioFire BCID2 panel no longer reports *mec*A/*mec*C for CoNS species except for Staphylococcus epidermidis and *S. lugdunensis*. Lastly, the BioFire BCID2 panel did misidentify some CoNS at the species level. However, this would not have a clinical impact, as different CoNS species are not treated differently at our institution.

In conclusion, the BioFire BCID2 panel is an accurate panel that is equivalent to the BioFire BCID panel compared to standard culture. The BioFire BCID2 panel offers several advantages over the BioFire BCID panel, including *Enterococcal* species identification, Gram-negative resistance gene detection, Salmonella identification, and the added *mec*A/*mec*C and MREJ target for better Staphylococcus aureus and CoNS differentiation. Most importantly, it provides additional clinical impact with the potential to decrease the time to optimal antimicrobial therapy compared to the BioFire BCID panel, with likely further impact at institutions with a higher prevalence of Gram-negative resistance.

## MATERIALS AND METHODS

### Study design and setting.

In this Colorado Multiple Institutional Review Board-approved study, we evaluated the BioFire blood culture identification 2 panel (BCID2; bioMérieux, BioFire Diagnostics, Salt Lake City, UT) simultaneously as a research-use-only prototype in parallel with the current standard of care for blood culture specimens at Children’s Hospital Colorado (CHCO) from 29 January 2020 to 1 August 2020. CHCO is the largest pediatric referral center for children in a 7-state region and includes a 434-bed acute care hospital in Aurora, Colorado, and 13 additional network locations offering outpatient, specialty, and urgent care. All sites use a common electronic health record (EHR; Epic Systems Corporation, Verona, WI).

### Laboratory methods.

All blood cultures received by the CHCO Microbiology Laboratory were included. The standard blood culture processing procedure in the CHCO Microbiology Laboratory was followed. Specimens were placed on the automated BacTec instrument (Becton, Dickinson and Co., Sparks, MD) and incubated. Once the specimens were positive, the microbiologist performed a Gram stain, placed them on agar plates, and processed the specimen on the BioFire blood culture identification panel (BCID; bioMérieux, BioFire Diagnostics, Salt Lake City, UT). A total of 191 positive blood culture specimens were also processed simultaneously on the BCID2. If a patient had multiple positive blood cultures, only the first blood culture was processed on BCID and BCID2, unless the subsequent culture revealed a different Gram stain. Any discrepant results between BCID2 and standard culture were resolved by performing 16S ribosomal PCR sequencing at Mayo Clinic Laboratories.

Blood culture and BCID results were reported to clinicians per our current standard of care at CHCO. From 8 a.m. to 5 p.m. Monday to Friday, results were called to the antimicrobial stewardship (ASP) team, who communicated results to the primary provider; otherwise, results were called to providers by the microbiology laboratory. Consensus pathogen-specific antimicrobial recommendations, based on national guidelines and local antibiogram data were created to standardize ASP recommendations (Table S1). The BCID2 was run in the background only, and therefore, results were not reported to providers.

### Chart abstraction.

Chart abstraction was performed on all patients via the EHR. Clinical and laboratory data were entered into standardized data collection forms developed in a standardized Research Electronic Data Capture (REDCap) database, hosted by the University of Colorado, Denver. EHR data included demographics, clinical history, the visit and admission encounter (if applicable), blood culture data, and antimicrobial data. The presence of an underlying medical condition was defined as a major system involvement for which the patient would be followed by a clinical specialist. The presence of an immunocompromising condition was defined as one of the following: oncology, bone marrow transplant, primary or acquired immunodeficiency, solid organ transplant, or chronic immunosuppressive medications. The time to BCID2 result was calculated from the time of specimen collection to the time that the BCID2 resulted on the instrument. The time to effective antimicrobial therapy was calculated from the time of specimen collection to the first dose of antimicrobial agent that was tested as susceptible according to Clinical and Laboratory Standards Institute criteria. The time to optimal antimicrobial therapy was calculated from the time of specimen collection to the time that the first dose of the optimal antimicrobial agent was received. Optimal antimicrobial therapy was defined based on predefined antimicrobial consensus recommendations developed in conjunction with our ASP team for each BCID2 target (Table S1), unless there was clinical justification for an alternative agent based on expert review (K.G. and S.R.D.). The time to theoretical optimal therapy was obtained by substituting the time to BCID2 result for the time to optimal therapy in patients with *Enterococcus* and CTX-M gene, as only these targets would have allowed for a change in antimicrobial therapy at the time of the BCID2 result.

### Statistical analysis.

Demographic and clinical characteristics were summarized using medians with ranges and proportions. Agreement between the organisms identified on BCID or BCID2 and standard culture was calculated as the proportion of blood cultures that matched for all target organisms, summarized with proportions and exact confidence intervals. Cultures with non-BCID and non-BCID2 targets were noted separately. Equivalence between BCID and BCID2 was tested using two one-sided tests considering an equivalence margin of 10%. Time to event outcomes were summarized using Kaplan-Meier estimators for the entire cohort and medians with interquartile ranges (IQRs) for patient subsets. For patients who were started on an effective or optimal antimicrobial regimen before test results were received, time was considered to be 0. If antibiotics were stopped because the clinical team considered the culture a contaminant, the event was censored at the stoppage time. If the time to effective or optimal therapy was never achieved, the event was censored at 14 days (336 h), as this was considered a complete course of therapy for bacteremia. The time to optimal therapy and time-to-theoretical optimal therapy were compared using a log-rank test. Within the Enterococcus faecalis subset, the differences in time to theoretical optimal therapy (time to BCID2 result) and time to optimal antimicrobial regimen were compared using a Wilcoxon signed-rank test. Significance was set at 0.05. R version 4.0.2 software (R Foundation for Statistical Computing, Vienna, Austria) was used for analysis.

### Data availability.

Data are available upon request.
