# Supplementary material for: Clinical Impact of the Expanded BioFire Blood Culture Identification 2 Panel in a U.S. Children’s Hospital
Source: Microbiol Spectr. 2021 Aug 25;9(1):10.1128/spectrum.00429-21. doi: 10.1128/spectrum.00429-21 (PMC8552780; doi:10.1128/spectrum.00429-21)
Supplement: SUPPLEMENTAL FILE 1 — Supplemental material. Download SPECTRUM00429-21_Supp_1_seq1.pdf, PDF file, 0.2 MB [file spectrum00429-21_supp_1_seq1.pdf]

Supplemental Table 1: Antimicrobial Stewardship Consensus Recommendations for BCID2 Targets

BCID2 Organism Targets Detected	BCID2 Resistance Targets Detected	Final Organism Identification Interpretation	First-line Antimicrobial Options (in order of preference)	Special Populations	Notes	ID consult
Enterococcus faecalis		Enterococcus faecalis (not VRE)	1) Ampicillin	•Consider adding gentamicin at synergistic dosing if concern for endocarditis<br>•*Clinical concern for active intraabdominal infectious process:* Continue gram negative and anaerobic coverage	•E. faecalis isolates at our institution are 100% ampicillin susceptible	
Enterococcus faecium		Enterococcus faecium (not VRE)	1) Vancomycin	• Consider adding gentamicin at synergistic dosing<br>• *Clinical concern for active intraabdominal infectious process:* Continue gram negative and anaerobic coverage		
Enterococcus faecalis or faecium	vanA/B pos	VRE	1) Linezolid or daptomycin	•*Oncology pt w/o lung disease:* daptomycin<br>• *Clinical concern for active intraabdominal infectious process:* Continue gram negative and anaerobic coverage		Rec
Gram positive cocci (BCID2 negative)			1) vancomycin	•*Oncology or GI patient:* Add Ampicillin (for possible E. gallinarum or E. cassileflavus)<br>•Consider daptomycin or adding clindamycin if history of leuconostoc or pediococcus (intrinsic vancomycin resistant)	•Possible organisms include: other Enterococcus species including gallinarum or casseliflavus (with intrinsic vancomycin resistance), Micrococcus, Rothia, Abiotrophia, (less commonly leuconostoc, pediococcus)	

Listeria monocytogenes		Listeria monocytogenes	1) Ampicillin	•*Meningitis, endocarditis, or immunocompromised*: ampicillin + gentamicin		Rec
Staphylococcus sp.	Note: no mecA will be reported	Coag Neg Staph	1) Vancomycin		•Can narrow off vancomycin once susceptibilities finalized<br>•Nafcillin causes vein irritation, best used continuous or with central line	
Staphylococcus epidermidis	mecA NOT detected	Staphylococcus epidermidis (without mec A)	1) Cefazolin (nafcillin for certain foci)	•*Endocarditis:* nafcillin 1st line although cefazolin is reasonable alternative, consider adding gentamicin<br>•*Endocarditis with prosthetic material:* add rifampin, gentamicin<br>•*CNS infection*: nafcillin	•Nafcillin causes vein irritation, best used continuous or with central line	
Staphylococcus lugdunensis	mecA NOT detected	Staphylococcus lugdunensis (without mec A)	1) Cefazolin (nafcillin for certain foci)	•*CNS infection*: nafcillin<br>•*Endocarditis:* consider adding gentamicin, nafcillin 1st line although cefazolin is reasonable alternative<br>•*Endocarditis with prosthetic material:* add rifampin, gentamicin	•Rare resistance to beta-lactams<br>•Can narrow to penicillin once susceptibilities finalized (if Beta-lactamase negative), reports of lower MICs to PCN if susceptible<br>•Nafcillin causes vein irritation, best used continuous or with central line	
Staphylococcus epidermidis or Staphylococcus lugdunensis	mecA pos	Coag Neg Staph (with mecA)	1)Vancomycin	•*Endocarditis:* consider adding gentamicin<br>•*Endocarditis with prosthetic material:* add rifampin, gentamicin		
Staphylococcus sp. + Staph aureus	mecA NOT detected	MSSA	1)Cefazolin (nafcillin for certain foci)	•*CNS infection:* nafcillin<br>•*Endocarditis:* consider adding gentamicin, nafcillin 1st line although cefazolin is reasonable alternative<br>•*Endocarditis with prosthetic material:* add rifampin, gentamicin	•Nafcillin causes vein irritation, best used continuous or with central line	Rec, if MSK

Staphylococcus sp. + Staph aureus	mecA pos	Mixed Coag Neg Staph (with mecA) + MSSA	1)Vancomycin	•*CNS infection or Endocarditis:* consider adding gentamicin<br>•*Endocarditis with prosthetic material:* add rifampin, gentamicin		Rec, if MSK
Staphylococcus sp. + Staph aureus	mecA pos AND MREJ pos	MRSA	1)Vancomycin<br>2) Ceftaroline, particularly for very ill patients at renal risk	•*CNS infection or Endocarditis:* consider adding gentamicin<br>•*Endocarditis with prosthetic material:* add rifampin, gentamicin<br>•*MSK infection:* consider ceftaroline first line		Rec, if MSK
Streptococcus sp.		Strep species (Not pneumococcus, GAS, GBS)	1)Vancomycin	• *Endocarditis*: consider adding gentamicin	•Narrow to ampicillin, penicillin, or cefazolin if susceptible<br>•Possible species include (but not limited to): Strep viridans group (including Strep mitis), Strep anginosus, alpha-Streptococcus, beta-Streptococcus (not A or B)	
Streptococcus sp. + Strep agalactiae		Group B Strep	1)Ampicillin	•*Neonates:* consider synergistic gentamicin for first 5 days		Rec
Streptococcus sp. + Strep pyogenes		Group A Strep	1) Ampicillin (or any beta-lactam)	•*Toxin ds:* consider adding clindamycin	•Can stop 2nd agent when improving	Rec
Streptococcus sp. + Strep pneumoniae		Pneumococcus	1)Ceftriaxone<br>2)Ampicillin	•*Meningitis*: Vancomycin + Ceftriaxone<br>•*Pneumonia:* High-Dose Ampicillin		
**BCID2 Organism Targets Detected**	**BCID2 Resistance Targets Detected**	**Final Organism Identification Interpretation**	**First-line Antimicrobial Options (in order of preference)**	**Special Populations**	**Notes**	**ID c/s?**

Acinetobacter baumannii		Acinetobacter baumannii	1)Meropenem	•High level resistance is rare at our center, so if patient is improving on cephalosporin may continue pending susceptibilities	•Often carries constitutive (not inducible) AmpC, so can rely on MIC<br>•Variable carbapenem-resistance mechanisms that are not detected (including efflux pumps and porin mutations)	Rec
Haemophilus influenza		Haemophilus influenza	1)Ceftriaxone		•Narrow to ampicillin if beta-lactamase neg. Beta-lactamase does not affect cephalosporins.	Rec
Neisseria meningitidis		Neisseria meningitidis	1)Ceftriaxone<br>2)Ampicillin		•May narrow once susceptibilities finalize	Rec
Pseudomonas aeruginosa	none	Pseudomonas aeruginosa	1)Cefepime<br>2)Meropenem	•*Very ill or immunocompromised:* meropenem or double coverage with beta-lactam PLUS (fluoroquinolone or aminoglycoside) during hi-inoculum period to assure at least one active agent	•Dose cefepime on high end, shortest interval (50mg/kg q8h) as intermediate MICs may be susceptible, dose-dependent<br>•Variable carbapenem-resistance mechanisms that are not detected (including efflux pumps and porin mutations)	Rec
Bacteroides fragilis		Bacteroides fragilis	1)Metronidazole	•*GI patients or GI abscess:* Plus ceftriaxone or cefepime (or single-agent meropenem)<br>• *immunocompromised:* Plus cefepime (or single-agent meropenem)		
Stenotrophomonas maltophilia		Stenotrophomonas maltophilia	1)TMP/SMX	•*Very ill or immunocompromised:* Dual coverage with TMP/SMX plus levofloxacin or ceftazidime until susceptibility results are known<br>•Consider aztreonam-avibactam (or aztreonam + ceftaz-avibactam) for	•Has intrinsic MBL (metallo-beta-lactamase), intrinsic resistance to carbapenems	Rec

				severely ill patients or those with MDR isolates.<br>•Could also consider eravacycline for MDR isolates		
Enteric bacteria		Enteric bacteria of species not listed below	1)Cefepime<br>2)Meropenem	•*GI abscess:* Add anaerobic coverage	•Potential for IBL<br>•Possible species include (but not limited to): Citrobacter spp, Enterobacter spp (other than cloacae), Cronobacter spp, Providencia spp, Yersinia spp, Serratia spp (other than marcescens), Morganella spp, Pantoea spp, Hafnia spp.	
Enteric bacteria + Enterobacter cloacae complex		Enterobacter cloacae	1)Cefepime<br>2)Meropenem	•*GI abscess:* Add anaerobic coverage	•Potential for IBL	
Enteric bacteria + Escherichia coli		E Coli or Shigella	1)Ceftriaxone	•*GI abscess:* Add anaerobic coverage<br>•*Concern for ESBL*: meropenem	•if on pressor support consider meropenem	
Enteric bacteria + Klebsiella oxytoca		Klebsiella oxyctoca	1)Ceftriaxone	•*GI abscess:* Add anaerobic coverage<br>•*Concern for ESBL*: meropenem	• if on pressor support consider meropenem	
Enteric bacteria + Klebsiella pneumoniae		Klebsiella pneumoniae	1) Ceftriaxone	•*GI abscess:* Add anaerobic coverage<br>•*Concern for ESBL*: meropenem	•if on pressor support consider meropenem	
Enteric bacteria + Klebsiella aerogenes		Klebsiella aerogenes	1) Cefepime	•Meropenem if critically ill	•High likelihood for IBL or ESBL (TEM-24, not CTX-M type)<br>•Dose cefepime on high end, shortest interval as intermediate MICs may be susceptible, dose-dependent	
Enteric bacteria + Proteus spp.		Proteus	1) Cefepime<br>2) Meropenem	•*GI abscess:* Add anaerobic coverage	•Potential for IBL (Indole pos species such as P. vulgaris or P. penneri)	

Enteric bacteria + Salmonella spp.		Salmonella	1) Ceftriaxone		•Requires higher dosing 75mg/kg/day	Rec
Enteric bacteria + Serratia marcescens		Serratia marcescens	1) Cefepime<br>2) Meropenem	•*GI abscess:* Add anaerobic coverage	•Potential for IBL	
Any Gram Negative Rod (except Pseudomonas and Acinetobacter)	*KPC pos	Carbapenem resistant gram-negative rod	1) Ceftazidime-avibactam<br>2) Meropenem-vaborbactam	•Consider aztreonam-avibactam if available (ceftolozane-tazobactam not as effective)	**•Call epi to notify and isolate**<br>•Note resistance to ceftaz-avi emerges on therapy (observed 10% of the time)	**Req**
Any Gram-Negative Rod (except Pseudomonas and Acinetobacter)	*IMP, NDM, or<br>VIM pos	MBL-producing gram negative rod	1) Aztreonam PLUS ceftazidime-avibactam	•Consider adding colistin/polymyxin B or tigecycline	**•Call epi to notify and isolate**<br>•Consider aztreonam-avibactam if available (ceftolazone-tazo and ceftaz-avi alone not shown to be as effective)	**Req**
Any Gram Negative Rod (except Pseudomonas and Acinetobacter)	*OXA-48-like	Carbapenem resistant gram-negative rod	1) Ceftazidime-avibactam		**•Call epi to notify and isolate**	**Req**
Any Gram Negative Rod	*CTX-M pos	ESBL-producer	1) Meropenem		•Call epi to notify and isolate	Rec
Any Gram Negative Rod	*Mcr-1 pos	Colistin-resistant	**Call ID**	Resistant to almost all antimicrobials, call ID and CDC for recommendations	**•Call epi to notify and isolate immediately**<br>•Typically found with additional resistance genes	**Req**
Pseudomonas aeruginosa	*KPC, IMP, Oxa-like, NDM, or VIM pos	Carbapenem-resistant Pseudomonas aeruginosa	1) Ceftolozane-tazobactam	Consider the following new agents if available:<br>•Impenem-relebactam<br>•Cifiderocol<br>Could consider colistin/polymyxin B (Note: tigecycline not effective)	**•Call epi to notify and isolate**<br>•Variable resistance mechanisms are not detected (including efflux pumps and porin mutations)	**Req**
Acinetobacter baumannii	*KPC, IMP, Oxa-like,	Carbapenem-resistant	1) Cifiderocol (if available)	Consider the following new agents if first-line agents not available:	**•Call epi to notify and isolate**	**Req**

	NDM, or VIM pos	Acinetobacter baumannii	2) Eravacycline (if available)	•Colistin/polymyxin B<br>•Tigecycline	•Note: Combination B-lactam/B-lactamase inhibitors are NOT reliable<br>•Variable resistance mechanisms are not detected (including efflux pumps and porin mutations)	

**BCID2 Organism Targets Detected/ Interpretation**	**First-line Antimicrobial Options (in order of preference)**	**Special Populations**	**Notes**	**ID consult**
Candida albicans	1) IV fluconazole	•*Extensive Azole Exposure:* IV micafungin<br>•*Neonate:* IV liposomal amphotericin B	•Discuss line removal.	Rec
Candida auris	1) IV micafungin	•*Ill or immunocompromised:* Consider dual IV micafungin + IV liposomal amphotericin B<br>•*Extensive Echinocandin Exposure:* IV liposomal amphotericin B<br>•*Neonate:* IV liposomal amphotericin B<br>•*CNS/Eye Disease*: IV liposomal amphotericin B	**•Call epi to notify and isolate immediately**<br>•High fluconazole resistance. Reports of isolates resistant to all classes<br>•Discuss line removal.	**Req**
Candida glabrata	1) IV micafungin	•*Extensive Echinocandin Exposure:* IV liposomal amphotericin B •*Neonate:* IV liposomal amphotericin B<br>•*CNS/Eye Disease*: IV liposomal amphotericin B	•Variable fluconazole resistance.<br>•Discuss line removal.	Rec
Candida krusei	1) IV micafungin	•*Extensive Echinocandin Exposure:* IV liposomal amphotericin B or IV voriconazole<br>•*Neonate:* IV liposomal amphotericin B<br>•*CNS/Eye Disease*: IV liposomal amphotericin B	•Intrinsic fluconazole resistance.<br>•Discuss line removal.	Rec
Candida parapsilosis	1) IV fluconazole	•*Extensive fluconazole exposure:* IV liposomal amphotericin B or IV Voriconazole<br>•*Neonate:* IV liposomal amphotericin B	•Higher MICs to echinocandins.<br>•Discuss line removal.	Rec
Candida tropicalis	1) IV fluconazole	•*Extensive fluconazole exposure:* IV micafungin<br>•*Neonate:* IV liposomal amphotericin B	•Discuss line removal.	Rec
Cryptococcus neoformans/gattii	1) IV liposomal amphotericin B	•CNS: IV liposomal amphotericin B +flucytosine<br>•Mild pulmonary disease or asymptomatic: Consider IV fluconazole		**Req**

Supplemental Table 2: non-BCID2 targets isolated in culture

Organism	Number isolated
Acinetobacter species	1
Micrococcus species	5
Rothia species	3
Corynebacterium	3
Moraxella species	1
Morganella species	1
Bacillus species	2
Abiotrophia species	2
Enterococcus gallinarum	1
